# 2119. *In Vitro* Activity of Aztreonam-Avibactam Against Enterobacterales Isolated from Pediatric and Adult Patients Collected During the ATLAS Global Surveillance Program, 2017-2021

**DOI:** 10.1093/ofid/ofad500.1742

**Published:** 2023-11-27

**Authors:** Mark Estabrook, Francis Arhin, Daniel F Sahm

**Affiliations:** IHMA, Schaumburg, Illinois; Pfizer, Inc., Kirkland, Manitoba, Canada; IHMA, Schaumburg, Illinois

## Abstract

**Background:**

The spread of antimicrobial resistance among clinically isolated Enterobacterales (Eba) threatens public health. Aztreonam (ATM) is a monobactam stable to hydrolysis by metallo-β-lactamases (MBLs) and avibactam (AVI) inhibits class A, class C, and some class D serine β-lactamases. ATM-AVI is being developed for use against infections caused by drug-resistant Eba, especially those co-producing MBLs and other β-lactamases. This study evaluated the *in vitro* activity of ATM-AVI and comparators against Eba collected in 2017-2021 from pediatric and adult patients as part of the ATLAS global surveillance program.

**Methods:**

84428 non-duplicate Eba isolates were collected from patients in 255 medical centers in 56 countries in Europe, Latin America, Asia/Pacific (excluding mainland China), and Middle East/Africa. Susceptibility testing was performed by CLSI broth microdilution and interpreted using CLSI 2023 breakpoints. PCR and sequencing were used to determine the β-lactamase genes present in all isolates with meropenem MIC >1 µg/mL, and a randomly sampled subset of approximately 80% of *Escherichia coli*, *Klebsiella* spp. and *Proteus mirabilis* with ATM or ceftazidime MIC >1 µg/mL.

**Results:**

MIC_90_ values for ATM-AVI of 0.12 µg/ml (pediatric isolates) and 0.25 µg/ml (adult isolates) were observed. Against all Eba isolates, ≤8 µg/ml of ATM-AVI was sufficient to inhibit 99.9% of both pediatric and adult isolates, whereas only 68.0% (pediatric) and 71.9% (adult) of these isolates were susceptible to ATM alone (table). Among isolates that screened positive for an MBL, MIC_90_ values for ATM-AVI were 0.5 µg/ml (pediatric) and 1 µg/ml (adult) and ATM-AVI inhibited 100% (pediatric) and 99.1% (adult) at concentrations ≤8 µg/ml. In contrast, only 16.1% (pediatric) and 17.9% (adult) of MBL-positive isolates were susceptible to ATM alone.

**Figure:**
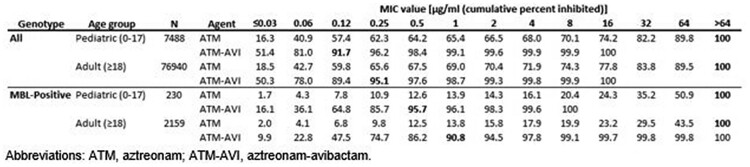

**Conclusion:**

Based on MIC_90_ values, ATM-AVI demonstrated potent *in vitro* activity against Eba isolated both from pediatric and adult patients. Avibactam’s ability to potentiate ATM against MBL-positive isolates warrants its continued development.

**Disclosures:**

**Mark Estabrook, PhD**, Pfizer Inc.: Honoraria **Daniel F. Sahm, PhD**, Merck & Co., Inc.: Honoraria|Pfizer Inc.: Honoraria|Venatorx: Paid fees for conducting the study and abstract preparation

